# Idiopathic Adult Ileocolic Intussusception Mimicking Cecal Carcinoma: A Case Report and Literature Review

**DOI:** 10.70352/scrj.cr.25-0030

**Published:** 2025-04-26

**Authors:** Rina Hashimoto, Tatsuo Kanda, Toshiyuki Saginoya, Masafumi Ishikawa, Hidetaka Kawamura, Yasushi Teranishi

**Affiliations:** 1 Department of Surgery, Southern TOHOKU General Hospital, Koriyama, Fukushima, Japan; 2 Department of Diagnostic Radiology, Southern TOHOKU General Hospital, Koriyama, Fukushima, Japan; 3 Department of Gastroenterology, Southern TOHOKU General Hospital, Koriyama, Fukushima, Japan; 4 Department of Minimally Invasive Surgery and Medical Oncology, Fukushima Medical University, Koriyama, Fukushima, Japan

**Keywords:** adult, idiopathic, intussusception, misdiagnosis, PET, pseudotumor

## Abstract

**INTRODUCTION:**

Adult intussusception is rare, accounting for approximately 5%–16% of all cases. Unlike pediatric intussusception, which is predominantly idiopathic, most adult cases are associated with organic lesions, nearly half of which are malignant. Idiopathic intussusception without a lead point is uncommon but appears to be increasingly recognized. We report a case of idiopathic adult ileocolic intussusception that mimicked cecal carcinoma in imaging studies.

**CASE PRESENTATION:**

A 63-year-old male with a history of gastric cancer recurrence presented with a 3-month history of abdominal pain. Contrast-enhanced computed tomography (CT) revealed circumferential thickening of the right colon, forming a “target sign,” and invagination of the ileocecal region into the right colon, suggesting ileocolic intussusception. Colonoscopy identified a nodular lesion presumed to be cecal carcinoma; however, the biopsy did not provide a definitive diagnosis of malignancy. Preoperative ^18^F-fluorodeoxyglucose-positron emission tomography (^18^F-FDG-PET/CT) revealed high FDG uptake at the leading edge of the intussusception; however, no findings indicative of metastatic disease were observed. The patient underwent elective surgery, and a right colectomy with lymph node dissection was performed. However, the intussusception was found to have spontaneously resolved at laparotomy. Histopathological examination showed mild intramural congestion in the ileocecal valve. Postoperative imaging confirmed the absence of any lead point lesion, resulting in a final diagnosis of idiopathic intussusception.

**CONCLUSIONS:**

This case highlights the diagnostic limitations of CT and PET/CT in evaluating lead points in adult intussusception, as false-positive findings are common. Given the possibility of spontaneous resolution, a conservative approach, including repeat imaging immediately before surgery, may be suitable in select cases of adult intussusception.

## Abbreviations

CT
: computed tomography
^18^F-FDG
: ^18^F-fluorodeoxyglucose
PET
: positron emission tomography

## INTRODUCTION

Intussusception is an acute abdominal condition characterized by the telescoping of a proximal segment of the intestine into the lumen of an adjacent distal segment, leading to bowel obstruction. The majority of cases occur in infants and children, while adult intussusception is rare, accounting for approximately 5%–16% of all cases.^1–3)^ Pediatric intussusception is typically idiopathic, occurring without an underlying organic cause. In contrast, most adult cases are associated with organic lesions that serve as a lead point, with nearly half of these lesions being malignant tumors.^1)^ As a result, surgical intervention is generally required for resolution in adult cases. However, although rare, there have been reports of idiopathic intussusception in adults without any underlying pathology. Interestingly, its incidence appears to be on the rise, likely due to the increased use of computed tomography (CT) imaging.^4)^ Consequently, there may be more cases where routine surgical intervention for adult intussusception is not always appropriate.

We report herein a case of ileocolic intussusception in a 63-year-old male with a history of recurrent gastric cancer. Various imaging modalities, including endoscopy, CT, and positron emission tomography (PET), suggested cecal carcinoma, leading to the decision to proceed with a right colectomy and lymph node dissection. However, surgical findings and further postoperative imaging revealed no causative lesion in the ileocecum, indicating that the intussusception was idiopathic.

This case was retrospectively considered an excessive surgical intervention. Along with this case, we review the clinical and imaging features that may help distinguish idiopathic intussusception in adults from cases caused by organic lesions, incorporating insights from the relevant literature.

## CASE PRESENTATION

A 63-year-old male presented with a 3-month history of persistent abdominal pain. He had previously undergone a total gastrectomy for stage III gastric adenocarcinoma at the age of 52. One year later, he developed para-aortic lymph node metastasis, which was effectively treated with 5 cycles of chemotherapy (S1 plus cisplatin), resulting in a long-term complete remission lasting 11 years.

**Fig. 1:**
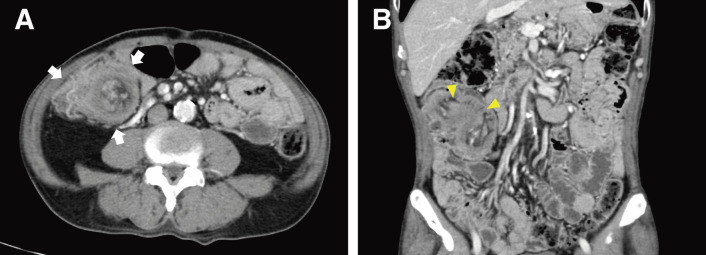
Contrast-enhanced CT scans. (**A**) The axial CT scan demonstrated a concentric, multilayered structure in the right colon (arrows), displaying the characteristic “target sign.” (**B**) The coronal CT image revealed an irregular mass within the hepatic flexure of the colon (arrowheads), accompanied by structures suggestive of mesenteric fat and vessels. CT, computed tomography

**Fig. 2:**
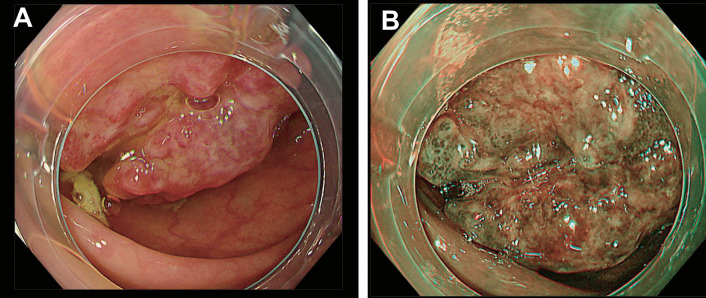
Endoscopic findings. Colonoscopy revealed that the terminal ileum had extended toward the hepatic flexure, displacing the cecum and the ascending colon upward. A large nodular mass formed the tip of the inverted ileocecal region, obscuring orifice identification. The tumor was endoscopically diagnosed as a Type 1 carcinoma of the cecum associated with intussusception. (**A**) Standard endoscopic view, and (**B**) narrow-band imaging view.

**Fig. 3:**
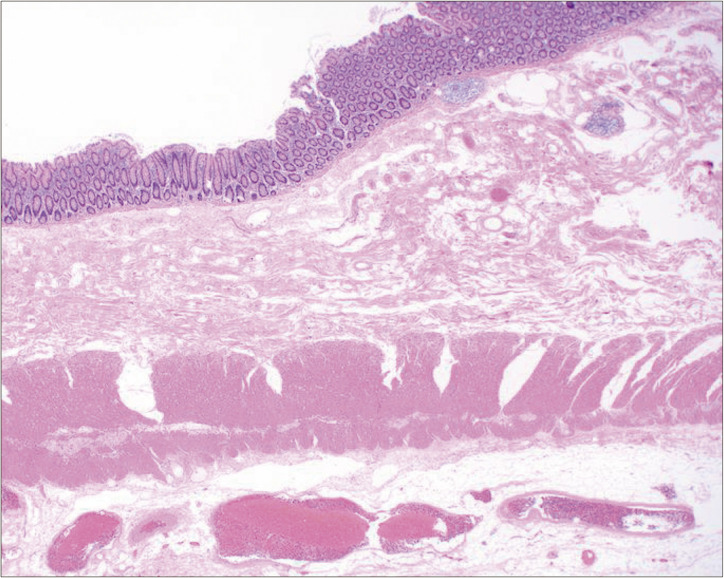
Histology of endoscopic biopsy specimens. The specimens obtained endoscopically from the lead point showed only mild submucosal congestion with minimal epithelial atypia (hematoxylin–eosin staining, original magnification ×20).

Despite unremarkable laboratory results, including tumor markers, a contrast-enhanced CT scan was performed due to clinical suspicion of a late recurrence of gastric carcinoma. The CT scan revealed circumferential wall thickening of the right colon, with mesenteric fat and vessels visible within it, forming the so-called “target sign.”^5)^ The coronal CT image demonstrated the ileocecal region invaginating into the right colon, strongly suggesting ileocolic intussusception (**Fig. 1**). Notably, there were no signs of bowel obstruction. A colonoscopy was scheduled 2 weeks later to further investigate the underlying cause of the intussusception. The colonoscopy confirmed the presence of intussusception extending into the hepatic flexure of the colon and identified a tumor as a lead point. The tumor presented as a large nodular mass with a shallow central depression (**Fig. 2**). However, endoscopic reduction of the intussusception was unsuccessful. The endoscopic findings strongly suggested intussusception secondary to cecal carcinoma, although the biopsy specimen showed only mild submucosal congestion with minimal epithelial atypia and was classified as Group 1 histologically (**Fig. 3**).

**Fig. 4:**
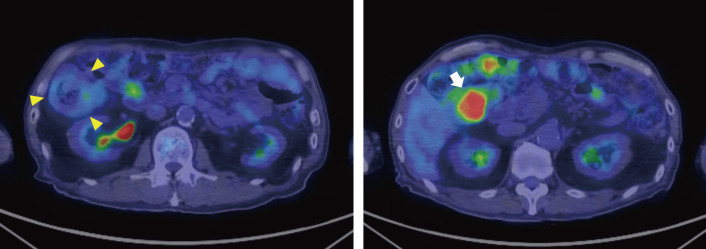
^18^F-fluorodeoxyglucose-positron emission tomography/computed tomography (^18^F-FDG-PET/CT). The CT image revealed a target sign in the right colon (arrowheads). A mass with high FDG uptake (maximum standardized uptake value, 17.2) was found within the colonic lumen on the anal side (arrow), suggesting that the tumor was the lead point of the intussusception. CT, computed tomography; ^18^F-FDG-PET/CT, ^18^F-fluorodeoxyglucose-positron emission tomography/computed tomography

Given that the tumor could be a metastasis from the prior gastric carcinoma, a preoperative ^18^F-fluorodeoxyglucose-PET/CT (^18^F-FDG-PET/CT) was performed. The ^18^F-FDG-PET/CT demonstrated a single mass with high FDG uptake at the lead point of the intussusception in the colon, with no other FDG-avid lesions suggestive of metastatic disease (**Fig. 4**). Based on these findings, a final diagnosis of ileocolic intussusception secondary to cecal carcinoma was made.

One month after the initial presentation, the patient underwent elective surgery with curative intent for the cecal carcinoma. Operative findings at laparotomy revealed that the intussusception had spontaneously resolved. An elastic mass was palpable in the ileocecal region, but no evident lymph node metastasis was noted. Given that the cecal carcinoma was considered stage I or II, an ileocolic resection with D2 lymph node dissection was performed.

**Fig. 5:**
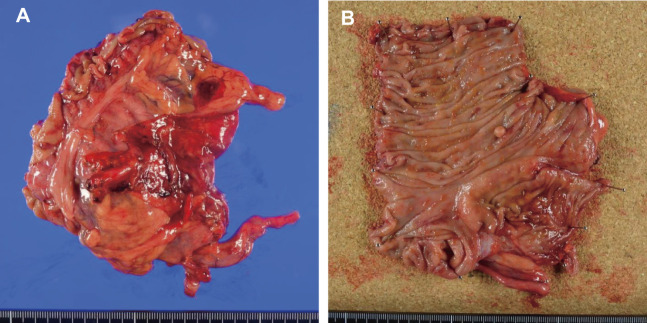
Gross appearance of the surgical specimen. (**A**) During surgery, it was found that the intussusception had spontaneously resolved, and no significant changes were observed on the serosal surface of the resected bowel. (**B**) Additionally, no tumor was found on the bowel mucosa, and only mild edema of the ileocecal valve was noted.

Pathological examination of the surgical specimen revealed no tumor in the excised bowel; the only histological finding was mild intramural congestion (**Fig. 5**). The elastic mass palpable during surgery was finally presumed to be due to an edematous ileocecal valve. Postoperative recovery was uneventful, and the patient was discharged on postoperative day 14. To rule out a missed lead point lesion, a contrast-enhanced CT was performed on postoperative day 9, followed by ^18^F-FDG-PET/CT 4 months later. Both scans revealed no significant tumor in the ileum or colon.

## DISCUSSION

We have reported a case of idiopathic ileocolic intussusception in a 63-year-old male patient. Unlike pediatric intussusception, which is predominantly idiopathic, adult intussusception often arises from underlying tumors. Therefore, surgical treatment, including bowel resection, is frequently employed in adult cases. Hong et al. conducted a systematic review of 40 studies that examined 1229 cases of adult intussusceptions.^4)^ They found that 32.9% of these cases were caused by malignant tumors, 37.4% by benign tumors, and only 15.1% were classified as idiopathic. Although idiopathic cases have been recognized as a minor etiology of adult intussusception, recent studies have shown that they account for 15% of cases, which is unexpectedly high.

In our case, preoperative evaluations, including colonoscopy, CT, and ^18^F-FDG-PET/CT, strongly suggested cecal carcinoma, which led to a right colectomy with lymph node dissection. However, postoperative findings confirmed idiopathic intussusception, indicating that the bowel resection was unnecessary. Given that spontaneous reduction was confirmed intraoperatively, a colotomy to verify the presence of carcinoma, followed by colopexy, might have been a reasonable alternative. Nevertheless, due to the increased risk of surgical site infection and the potential for relapse from undetected lesions, modifying the surgical approach intraoperatively was challenging. This case highlights the diagnostic challenges in differentiating idiopathic intussusception from tumor-associated cases, raising the question of whether idiopathic intussusception should have been more strongly suspected before surgery.

The patient had a 3-month history of abdominal pain, indicative of a chronic course, which can be considered a notable clinical feature. Early studies have shown that abdominal pain, nausea, and vomiting are the most common symptoms of adult intussusceptions.^1,6)^ Additionally, although subacute or chronic presentations were not widely recognized in the past, they are now increasingly understood as common features of adult intussusception, setting adult cases apart from pediatric ones. A retrospective study of 196 adult intussusception cases over 26 years found a median interval of 69 days (range: 1 day–3 years) from symptom onset to diagnosis.^7)^ Another study reported a median duration of 18 days (range: 1–365 days).^8)^ These findings suggest that chronic symptomatology is a consistent characteristic of adult intussusception, regardless of the presence of an organic lesion.

Adult intussusception is commonly classified by anatomical location into enteric, ileocolic, and colonic types. According to Hong et al.’s review,^4)^ the frequencies are 49.5% for the enteric type, 29.1% for the ileocolic type, and 19.9% for the colonic type. Their review also detailed the prevalence of idiopathic cases within each type: 23.5% for the enteric type, 23.0% for the ileocolic type, and 20.0% for the colonic type. Our case was categorized as ileocolic intussusception, wherein the distal ileum invaginated into the ascending colon. Unlike pediatric cases, idiopathic adult intussusception does not appear to involve the ileocolic type preferentially. Consequently, idiopathic adult intussusception lacks distinctive clinical or anatomical features, making preoperative suspicion difficult.

Advancements in CT imaging have enabled more detailed evaluation of bowel pathology, including intussusception. Tresoldi et al. evaluated the capability of multidetector CT to differentiate intussusception with and without a lead point in 93 adult patients.^9)^ In their study, 2 board-certified radiologists independently and blindly reviewed the CT images. Of the 93 cases, Radiologist 1 identified a lead mass in 27 patients (29%), whereas Radiologist 2 identified it in 19 patients (20%). However, the positive predictive values were low at 37% and 47%, respectively, indicating a substantial rate of false positives. Their study also highlighted imaging differences between intussusceptions with and without a lead point. Intussusceptions with no lead points generally had shorter lengths (4.0 vs. 8.9 cm and 4.9 vs. 11.1 cm) and smaller axial diameters (2.8 vs. 4.4 cm and 3.0 vs. 4.8 cm) than those with lead points. Furthermore, the non-lead point cases were less likely to exhibit signs of bowel obstruction or paraenteric infiltration than the lead point ones. In our case, the intussusception measured 9.4 cm in length and 5.7 cm in diameter, with paraenteric infiltration present. These features matched 3 of the 4 imaging characteristics proposed for lead point intussusceptions, except for the absence of bowel obstruction. Because Tresoldi et al.’s study included a significant number of incidentally detected enteric-type intussusceptions, their findings regarding non-lead point intussusceptions may reflect characteristics specific to incidentally detected enteric-type cases rather than those of non-lead point intussusception in surgical practice. Although multidetector CT characteristics can provide diagnostic clues, they are not definitive for distinguishing idiopathic intussusception from tumor-associated cases.

The patient in our case underwent ^18^F-FDG-PET/CT to rule out distant metastasis, given his history of complete response after gastric cancer recurrence. The PET/CT scan showed intense FDG uptake in the intussuscepted segment, strongly suggesting the presence of cecal carcinoma and supporting a preoperative diagnosis of intussusception associated with malignancy. However, the high FDG uptake was ultimately attributed to ischemia or inflammation in the intussuscepted segment, as follow-up PET/CT revealed no significant FDG-avid lesion.

To investigate PET findings in adult intussusception, we searched the PubMed database using 2 keywords, “adult intussusceptions” and “positron emission tomography/PET.” As of December 2024, this search identified 22 studies. We excluded 2 studies published in languages other than English and 7 studies that did not report preoperative PET/CT findings, leaving 13 eligible cases for analysis (**Table 1**).^10–22)^ Among these, 11 cases were associated with malignant tumors, 1 with a benign tumor, and 1 with a non-neoplastic lesion. Most PET/CT scans were performed to evaluate tumor progression rather than to determine the cause of intussusception. Our literature review revealed no data on PET findings specific to idiopathic intussusception, making its role in differentiating malignant cases from idiopathic ones unclear.

**Table 1:** Summary of patients with intussusception who underwent PET/CT

Age/sex	Symptoms and signs	Causative disease	Type	Indication for PET/CT	PET/CT findings	Reference no.
71/M	Abdominal pain	Adenocarcinoma of the transverse colon	Colonic	Staging small cell lung cancer	A mass with intense FDG uptake in the transverse colon	^10)^
49/F	Abdominal pain	Metastatic melanoma	Details unknown	Not mentioned	Intense FDG uptake at the apex of the intussusception	^11)^
53/M	Fever, anemia, and vomiting	Metastatic melanoma	Enteric	To further evaluate the metastatic lesions	Multiple small bowel intussusceptions with hypermetabolic areas on fused image	^12)^
55/F	Abdominal pain	Aggressive fibromatosis	Ileocolonic	To evaluate possible malignancy	Intense FDG uptake in the region of the cecum and ascending colon, SUVmax 5.4	^13)^
74/M	Abdominal pain, vomiting, and constipation	Metastatic lung cancer	Enteric	Diagnostic workup of a lung mass	Intense FDG uptake in the left abdomen, SUVmax 11.4	^14)^
30/F	Abdominal pain	Inflammatory pseudotumor	Colonic	Not mentioned	A mass with intense FDG uptake in the descending colon, SUVmax 7.8	^15)^
55/M	Upper abdominal discomfort	Non-Hodgkin lymphoma	Ileocolonic	To evaluate the extent of disease	Intense FDG uptake in the right abdomen	^16)^
82/M	Abdominal pain, nausea, and fatigue	Non-Hodgkin lymphoma	Enteric	To evaluate the extent of disease	Hypermetabolic mass in the small bowel and hypermetabolic lymph nodes in the mesenteric root	^17)^
49/F	Right abdominal discomfort and constipation	Mantle cell lymphoma	Colonic	To evaluate the extent of disease	Intense FDG uptake, SUVmax 7.7	^18)^
55/F	Nausea, epigastric pain, abdominal distention, and anemia	Metastatic melanoma	Enteric	To assess the extent of cancer spread	Hypermetabolic mass in the small bowel	^19)^
72/M	Anemia	Carcinoma of the small bowel	Enteric	To assess the stage of the suspected lung cancer	Intense FDG uptake in the small bowel, SUVmax 9.2	^20)^
89/M	No symptom or sign	Metastatic giant cell tumor	Enteric	To evaluate the extent of disease	Intense focal FDG uptake in small bowel mass	^21)^
47/M	No symptom or sign	Metastatic non-clear cell renal cell carcinoma	Enteric	Follow-up for renal cell carcinoma	FDG uptake in the segment of small bowel involved in the intussusception	^22)^

FDG, fluorodeoxyglucose; PET/CT, positron emission tomography/computed tomography; SUVmax, maximum standardized uptake value

In the present case, intraoperative findings showed spontaneous resolution of the intussusception, underscoring the need for greater caution in management. As the use of CT continues to increase, there is a growing trend in the incidental diagnosis of intussusceptions.^4)^ Rea et al. analyzed 170 cases of intussusception diagnosed by CT and found that only 30 patients (17.6%) underwent surgery; in half of these, the intussusception had spontaneously resolved intraoperatively.^23)^ Similarly, a study of 318 adult intussusception patients reported that only 40% required surgery, while the remaining 60% were managed conservatively.^24)^ These findings suggest that routine surgical intervention should be reconsidered in favor of more conservative approaches, particularly in non-obstructive and non-emergent cases. They also indicate that a more cautious strategy, including repeat imaging immediately before surgery, may be warranted in selected cases. Although no evidence-based criteria exist, repeat imaging may be reasonable in patients with symptom improvement or when a significant interval has elapsed since the initial CT scan.

## CONCLUSIONS

We have presented a case of adult idiopathic intussusception. Despite imaging findings strongly suggesting cecal carcinoma, histopathological examination revealed no tumor. Our literature review indicated that no definitive criteria based on symptoms, anatomical location, or CT findings reliably distinguish idiopathic from tumor-associated intussusception, underscoring the diagnostic limitations of CT and PET/CT. Considering the possibility of spontaneous resolution, a more cautious approach, including repeat imaging immediately before surgery, may be advisable in managing adult intussusception. Further studies are needed to establish criteria for identifying patients who may benefit from additional imaging.

## ACKNOWLEDGMENTS

The authors thank Dr. Noriyuki Uesugi for his invaluable assistance with the pathological diagnosis in this study.

## DECLARATIONS

### Funding

No funding was received.

### Authors’ contributions

TK and YT were the attending physicians and jointly conceptualized the study.

RH and TK drafted the original manuscript.

RH, MI, and TS prepared the images used in the manuscript and contributed their expertise to the drafting process.

RH, TS, and HK cooperatively conducted a literature review.

HK assisted in manuscript revision.

YT oversaw the administration of the project.

All authors have read and approved the final version of the manuscript.

### Availability of data and materials

The datasets used and/or analyzed in this study are available from the corresponding author on reasonable request.

### Ethics approval and consent to participate

Not applicable.

### Consent for publication

Written informed consent was obtained from the patient for the publication of the case report and all accompanying images.

### Competing interests

The authors declare that they have no competing interests.
